# Computational methods, clinical evidence, and translational readiness for artificial intelligence for HER2 assessment using silver in situ hybridization (SISH)

**DOI:** 10.1186/s13058-026-02363-z

**Published:** 2026-08-20

**Authors:** Wael Badawy

**Affiliations:** Faculty of Artificial Intelligence, Egyptian Russian University, Badr City, Cairo Egypt

**Keywords:** HER2, Silver in situ hybridization, SISH, Artificial intelligence, Digital pathology, Deep learning, Breast cancer

## Abstract

Accurate determination of human epidermal growth factor receptor 2 (HER2) gene amplification is a cornerstone of precision oncology in breast cancer and an expanding range of solid tumors. While immunohistochemistry (IHC) remains the first-line screening modality, in situ hybridization (ISH) techniques are essential for resolving equivocal cases and providing quantitative assessment of HER2 gene status. Among ISH methods, silver-enhanced in situ hybridization (SISH) has emerged as a clinically relevant bright-field alternative to fluorescence in situ hybridization (FISH), offering permanent staining, high concordance, and compatibility with routine histopathology workflows. Despite these advantages, SISH interpretation remains labor-intensive and subject to interobserver variability, particularly in borderline and heterogeneous tumors. The rise of digital pathology and artificial intelligence (AI) has enabled automated analysis of whole-slide images (WSIs), supporting tasks such as nuclei segmentation, signal detection, HER2 copy-number estimation, and HER2/CEP17 ratio computation. However, current evidence remains heterogeneous, with limitations including small datasets, limited external validation, and insufficient methodological transparency. This systematic review synthesizes current literature on AI-assisted HER2 assessment with a specific focus on SISH and related bright-field ISH modalities. We examine computational workflows, validation strategies, performance benchmarks, and translational readiness while critically appraising limitations in the evidence base. The analysis demonstrates that AI can support reproducible and scalable HER2 assessment when aligned with clinical guidelines, but emphasizes that clinical deployment requires rigorous validation, uncertainty-aware design, and integration with expert pathology workflows.

## Introduction

The identification of HER2 amplification has fundamentally transformed the management of breast cancer by enabling targeted therapeutic strategies that significantly improve patient outcomes. Since the introduction of HER2-targeted therapies, accurate determination of HER2 status has become a mandatory component of diagnostic evaluation. Current clinical guidelines recommend immunohistochemistry as the initial screening approach, followed by in situ hybridization for equivocal cases in order to confirm gene amplification and establish definitive HER2 status [6, 10].

Silver-enhanced in situ hybridization occupies a distinct and increasingly important position within the spectrum of HER2 testing modalities. Unlike fluorescence-based ISH techniques, which require specialized equipment and are susceptible to signal fading, SISH produces stable metallic silver deposits that can be visualized under standard bright-field microscopy. This allows pathologists to evaluate HER2 gene amplification in direct conjunction with tissue morphology, which is particularly valuable in heterogeneous tumors. Multiple comparative studies have demonstrated concordance rates between SISH and FISH exceeding 95%, supporting its reliability as a clinically acceptable alternative for HER2 testing [2, 16].

However, SISH interpretation is inherently complex and cognitively demanding. The diagnostic process requires accurate identification of invasive tumor nuclei, enumeration of HER2 and CEP17 signals, calculation of the HER2/CEP17 ratio, and evaluation of the average HER2 copy number per cell. These tasks are further complicated by variability in staining quality, signal clustering, and the presence of heterogeneous tumor regions. In particular, intratumoral heterogeneity may result in focal amplification patterns that are difficult to detect through manual assessment alone.

Although recent clinical attention has focused on HER2-low and HER2-ultralow categories, it is important to clarify that these classifications are defined by immunohistochemical expression patterns rather than by ISH-based measurements. Consequently, SISH should not be presented as a direct determinant of HER2-low or ultralow status, but rather as a complementary method that may support integrated diagnostic workflows in selected cases [9, 19].

In parallel with these developments, digital pathology has undergone rapid transformation with the widespread adoption of whole-slide imaging technologies. Advances in artificial intelligence, particularly deep learning architectures such as convolutional neural networks and vision transformers, have enabled automated analysis of histopathological images with unprecedented scale and consistency. AI systems have demonstrated strong performance across a range of pathology tasks, including tumor detection, grading, and biomarker quantification [12, 18].

**Fig. 1:**
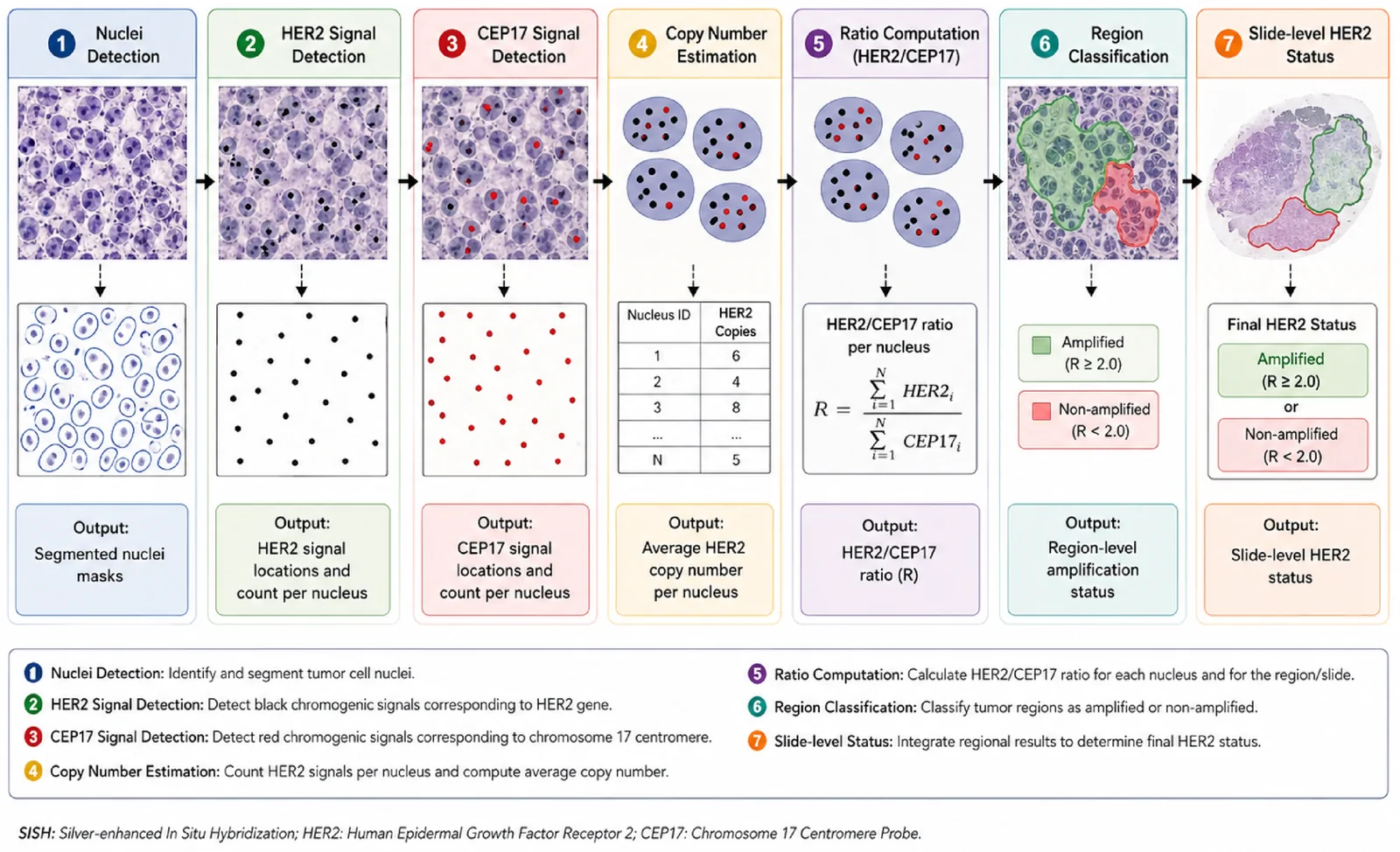
Schematic illustration of AI tasks in HER2 bright-field ISH analysis

The conceptual role of artificial intelligence in HER2 bright-field ISH analysis, including nuclei segmentation, signal detection, and quantitative ratio computation, is illustrated in Fig. 1.

Despite significant progress in AI-assisted pathology, most existing studies have focused on IHC and fluorescence-based ISH modalities, with relatively limited attention given to SISH-specific challenges. This review addresses this gap by providing a comprehensive synthesis of AI applications in HER2-SISH assessment, with emphasis on methodological rigor, clinical relevance, and translational readiness.

The remainder of this paper is structured as follows. Section “Methods: systematic review design” describes the systematic review methodology, including the PRISMA-based study selection process, search strategy, and inclusion criteria. Section “Technical foundations of HER2-SISH image analysis” presents the technical foundations of HER2-SISH image analysis and discusses the distinctive characteristics of SISH for artificial intelligence applications. Sections “AI for nuclei detection and segmentation in HER2-SISH” and “Automated HER2 and CEP17 signal detection” review AI-based approaches for nuclei segmentation, signal detection, HER2 copy-number estimation, and HER2/CEP17 ratio computation. Section “Region-level and whole-slide classification approaches” discusses region-level and whole-slide classification approaches, while Section “Integration with other HER2 modalities” examines the integration of AI-assisted HER2 assessment across IHC, SISH, DISH, and FISH modalities. Section “Results synthesis: study-by-study mapping (continued and deepened)” synthesizes the reported results and comparative performance findings. Sections “Tumor heterogeneity and focal amplification” and “Sources of error and failure modes in HER2-SISH AI systems” analyze tumor heterogeneity, focal amplification, and major sources of error and failure modes in AI-assisted HER2 assessment. Section “Critical appraisal of current evidence” and “Reproducibility, data availability, and benchmarking” critically evaluate the current evidence base, reproducibility challenges, and benchmarking limitations. Section “Regulatory landscape and translational readiness” to “Clinical implications and workflow integration” discuss translational readiness, regulatory considerations, explainability, ethical implications, and clinical workflow integration. Section “Limitations of the current evidence base” outlines the principal limitations of the current literature, while Section “Future directions and research priorities” highlights future research directions and emerging opportunities. Finally, Section “Conclusion” concludes the paper and summarizes the key findings and translational implications of AI-assisted HER2 assessment using silver-enhanced in situ hybridization.

## Methods: systematic review design

This systematic review was conducted in accordance with the PRISMA 2020 framework, adapted to accommodate the methodological diversity inherent in computational pathology research. The primary objective was to identify and synthesize peer-reviewed literature addressing artificial intelligence applications in HER2 assessment using SISH or closely related bright-field ISH modalities.

A comprehensive literature search was performed across PubMed, Scopus, Web of Science, and IEEE Xplore using combinations of keywords related to HER2, silver in situ hybridization, bright-field ISH, and artificial intelligence. The final search date should be explicitly reported to ensure reproducibility. Additional studies were identified through manual screening of reference lists from relevant articles and reviews.

Studies were included if they employed machine learning or deep learning methods and addressed HER2 assessment using SISH, dual-color bright-field ISH, or related modalities, while reporting quantitative performance metrics. Studies focusing exclusively on IHC, H&E, or fluorescence-based methods were excluded unless they provided methodological insights relevant to SISH workflows. Studies such as Qaiser et al. [12] were included for comparative context but not classified as direct HER2-SISH evidence.

The study selection process involved a two-stage screening procedure consisting of title and abstract review followed by full-text assessment. Extracted data included dataset characteristics, AI architecture, task definition, validation strategy, performance metrics, and clinical reference standards.

**Fig. 2:**
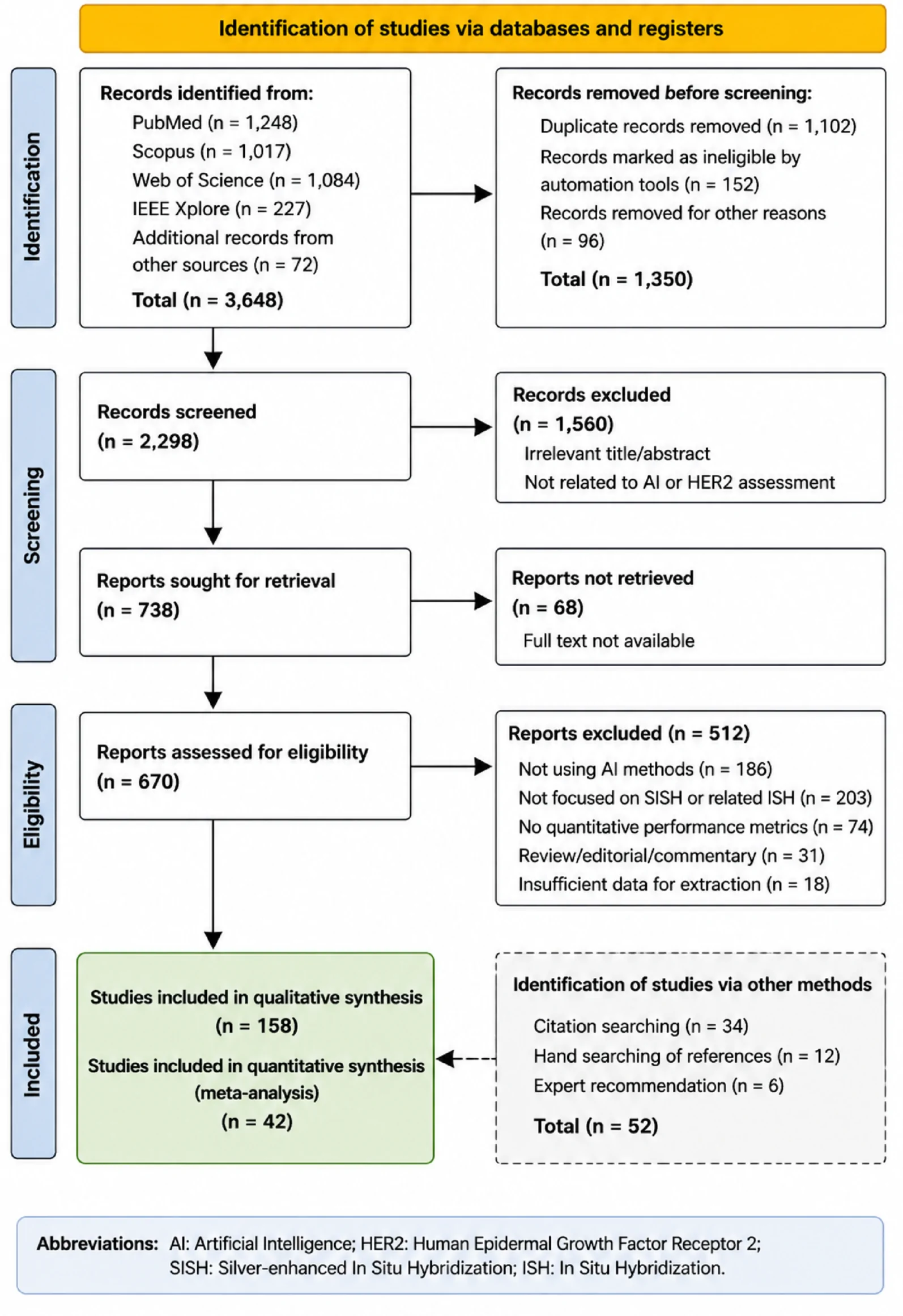
PRISMA 2020 flow diagram summarizing study identification, screening, eligibility assessment, and final inclusion

The study identification, screening, eligibility assessment, and inclusion process is illustrated in Fig. 2.

A structured critical appraisal was incorporated into the methodology to evaluate dataset size, diversity, external validation, ground truth definition, and clinical alignment. This approach was adopted to address limitations observed in prior studies, particularly the tendency to report technical performance metrics without sufficient evaluation of clinical validity and generalizability.

## Technical foundations of HER2-SISH image analysis

SISH produces discrete metallic signals corresponding to HER2 gene amplification within a hematoxylin-counterstained tissue background. While this enables simultaneous evaluation of molecular signals and morphological context, it also introduces computational challenges related to signal clustering, variable staining intensity, and background artifacts.

**Fig. 3:**
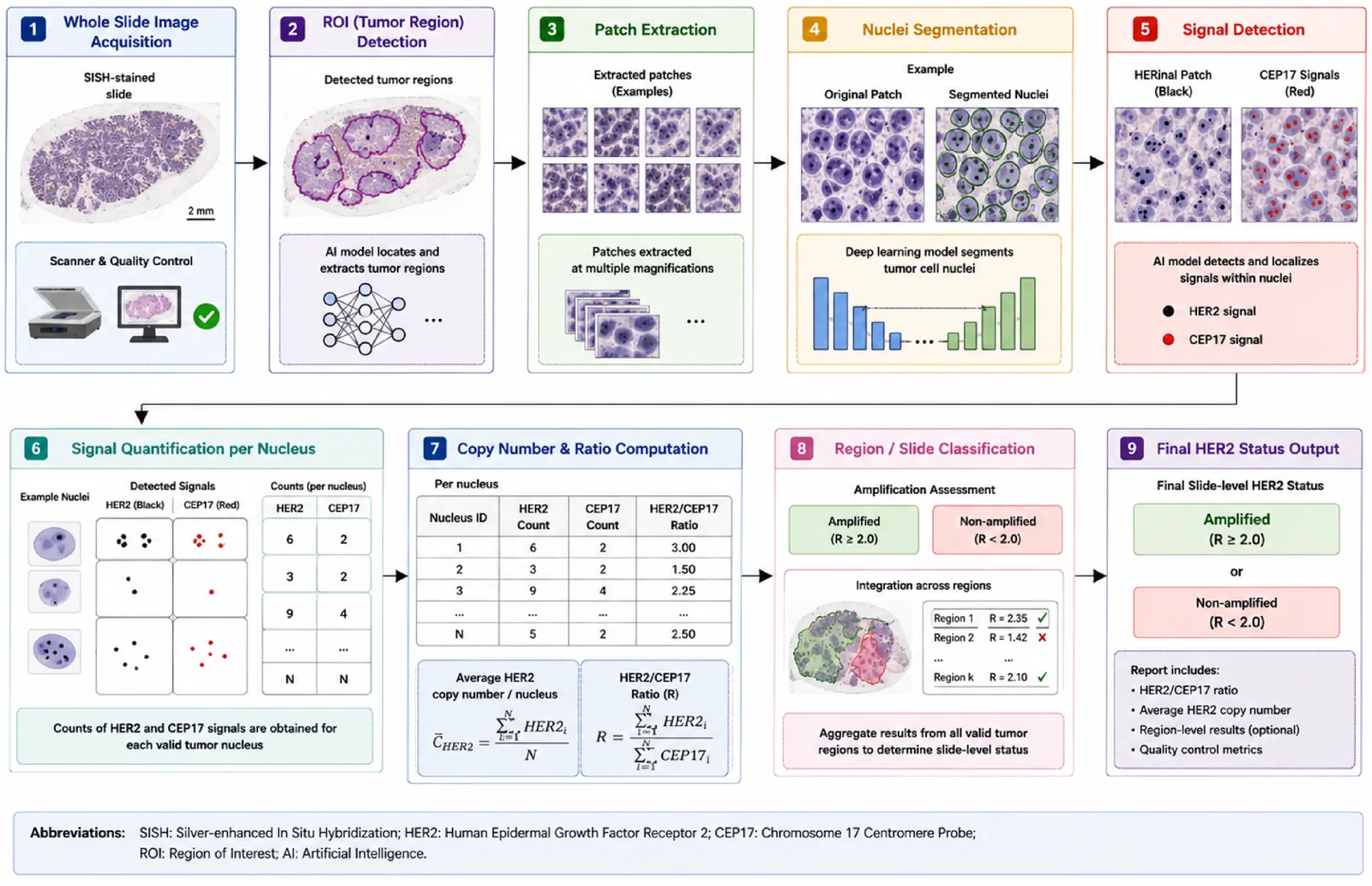
End-to-end HER2-SISH computational pipeline

From a computational perspective, HER2-SISH analysis typically follows a structured workflow beginning with whole-slide image acquisition and quality control, followed by tumor region identification, nuclei segmentation, signal detection, copy-number estimation, and HER2/CEP17 ratio computation. The complete computational pipeline is illustrated in Fig. 3.

Errors introduced at early stages of this pipeline may propagate through subsequent steps and affect final classification outcomes. Consequently, robust design of segmentation and detection models is essential for reliable HER2 assessment [13].

SISH differs from other HER2 testing modalities in that it combines direct gene-level quantification with bright-field morphological context. Compared with immunohistochemistry, it provides quantitative measurement of gene amplification rather than protein expression. Compared with fluorescence-based ISH, it offers permanent signals and compatibility with routine histopathological workflows. These features make SISH particularly suitable for explainable AI approaches, in which intermediate outputs such as detected nuclei, counted signals, and computed ratios can be visualized and interpreted by pathologists.

The progression of artificial intelligence tasks in HER2-SISH analysis, from low-level image processing to clinically actionable outputs, is summarized in Table 1. Table 1 summarizes the principal AI task categories addressed in contemporary HER2 assessment research, highlighting the progression from low-level image analysis (nuclei and signal detection) to clinically actionable outputs (HER2/CEP17 ratio, HER2-low classification). Signal-based approaches demonstrate the strongest alignment with clinical guidelines and regulatory expectations, whereas classification-based methods offer computational efficiency at the cost of interpretability. Multimodal workflows integrating IHC and ISH increasingly reflect real-world diagnostic practice.

**Table 1:** Core artificial intelligence tasks in HER2 assessment using SISH and related In situ hybridization modalities

AI task	Primary modality	Description of task	Typical performance range	References
Nuclei detection and segmentation	HER2-SISH WSI	Instance-level segmentation of tumor nuclei to enable downstream signal quantification and ratio computation	F1-score ≈ 0.97–0.98	Rehman et al. [14], Qaiser et al. [12]
HER2 signal detection	SISH/Dual ISH	Detection and counting of HER2 gene signals within segmented nuclei	Precision > 0.90; high concordance	Rehman et al. [13], Shi et al. [15]
CEP17 signal detection	SISH/Dual ISH	Identification of chromosome 17 control signals for normalization	Precision > 0.90	Shi et al. [15]
HER2/CEP17 ratio computation	Bright-field ISH	Automated calculation of amplification ratio in accordance with ASCO/CAP guidelines	Pearson r ≈ 0.9; > 95% concordance	Rehman et al. [13], Papouchado et al. [10]
Patch-level classification	HER2-SISH	Classification of image patches as amplified vs non-amplified using CNNs or ViTs	Accuracy ≈ 87–99% (patch level)	Tan et al. [17]
Whole-slide image (WSI) classification	HER2-SISH	Aggregation of patch predictions to infer slide-level HER2 status	Accuracy ≈ 79–98%	Tan et al. [17], Qaiser et al. [12]
Multimodal HER2 assessment	IHC + ISH	Integration of AI-assisted IHC scoring with ISH confirmation workflows	Near-expert agreement	Palm et al. [9], Wu et al. [19]
HER2-low/ultralow detection	IHC ± ISH	Improved discrimination of low-expression categories relevant to ADC therapies	Increased sensitivity, higher kappa	Wu et al. [19],Albuquerque et al. [1]
Uncertainty estimation/QC	SISH/IHC	Identification of low-confidence cases for human review	Emerging	Jung et al. [8], Malinowski et al. [3]

A comparative overview of HER2 testing modalities, including IHC, SISH, dual-color bright-field ISH, and FISH, is provided in Table 2. Table 2 contrasts HER2 testing modalities through the lens of artificial intelligence readiness and clinical utility. While IHC benefits from extensive AI research and plays a critical role in HER2-low detection, SISH uniquely combines bright-field interpretability with direct gene-level quantification, making it particularly well suited for explainable, guideline-aligned AI workflows. Compared with FISH, SISH offers advantages in slide permanence and morphological context, albeit with greater sensitivity to scanning quality. Collectively, these characteristics position SISH as a promising but still maturing substrate for AI-assisted HER2 diagnostics.

**Table 2:** Comparison of HER2 testing modalities with respect to artificial intelligence suitability and clinical utility

Dimension	Immunohistochemistry (IHC)	Silver in situ hybridization (SISH)	Fluorescence in situ hybridization (FISH)
Primary clinical role	Initial HER2 screening	Confirmatory gene amplification testing	Reference confirmatory testing
Signal type	Membranous protein staining	Metallic silver DNA signals (HER2, CEP17)	Fluorescent DNA signals
Imaging modality	Bright-field microscopy	Bright-field microscopy	Fluorescence microscopy
Slide permanence	Permanent	Permanent	Non-permanent (signal fading)
Morphological context	Excellent	Excellent	Limited
Manual scoring complexity	Moderate–high (subjective)	High (signal counting)	High (signal counting)
Interobserver variability	Moderate–high	Low–moderate	Low–moderate
AI task maturity	High (well-studied)	Moderate–high (emerging)	High
AI nuclei detection performance	High	Very high (F1 ≈ 0.97–0.98)	High
AI signal detection robustness	Moderate (membrane ambiguity)	High (distinct dots)	Very high (high contrast)
HER2/CEP17 ratio automation	Not applicable	Direct, guideline-aligned	Direct, guideline-aligned
Suitability for HER2-low detection	High (with AI assistance)	Supportive (confirmatory)	Limited
Sensitivity to scan quality	Moderate	High	High
Multicenter AI generalizability	Demonstrated	Emerging	Demonstrated
Regulatory readiness for AI	Advanced	Advancing	Advanced
Cost and infrastructure	Low–moderate	Moderate	High
AI explainability	Moderate	High (signal-based)	High
Typical AI clinical role	Decision support	Decision support/confirmation	Decision support/confirmation

While SISH offers advantages in interpretability and morphological context, it remains sensitive to variations in scanning quality, staining protocols, and signal morphology, which may influence AI performance [3].

Table 3 summarizes the principal algorithmic strategies employed in AI-based HER2 assessment, highlighting the methodological diversity across segmentation, detection, classification, and multimodal integration. Signal-based pipelines combining deep learning with rule-based aggregation offer the strongest alignment with clinical guidelines and explainability requirements, particularly in SISH workflows. Classification-based and weakly supervised approaches provide scalability and reduced annotation costs but require careful validation to ensure clinical reliability.

**Table 3:** Algorithmic approaches and model architectures used in AI-based HER2 assessment

AI approach	Typical model architectures	Primary input	Main output	Strengths	Limitations	References
Nuclei instance segmentation	StarDist, mask R-CNN, U-Net variants	SISH WSI or ROI patches	Individual tumor nuclei masks	High accuracy in dense tissue; essential for downstream tasks	Sensitive to focus and staining variation	Rehman et al. [14], Qaiser et al. [12]
Signal (dot) detection	CNN-based object detectors, blob detectors + DL refinement	Segmented nuclei regions	HER2 and CEP17 signal coordinates	Direct biological interpretability	Struggles with clustered or overlapping signals	Rehman et al. [13], Shi et al. [15]
Signal quantification & ratio computation	Rule-based aggregation + DL detection	Dot counts per nucleus	HER2/CEP17 ratio	Guideline-aligned; explainable	Ratio instability in low-signal cases	Rehman et al. [13], Papouchado et al. [10]
Patch-level classification	CNNs (ResNet, DenseNet), Vision Transformers (ViT)	SISH image patches	Amplified vs non-amplified label	Fast inference; robust to local noise	Limited interpretability; indirect clinical mapping	Tan et al. [17]
Whole-slide aggregation	Majority voting, attention pooling, rule-based fusion	Patch-level predictions	Slide-level HER2 status	Handles heterogeneity	Sensitive to aggregation strategy	Tan et al. [17], Qaiser et al. [12]
Multimodal learning	Late fusion CNNs, hybrid pipelines	IHC + ISH / H&E + ISH	Integrated HER2 status	Improves borderline case accuracy	Increased system complexity	Palm et al. [9], Wu et al. [19]
Weakly supervised learning	Multiple-instance learning (MIL)	WSI with slide labels	Slide-level prediction	Reduces annotation burden	Lower spatial precision	Qaiser et al. [12]
Uncertainty-aware models	Conformal prediction, Bayesian DL	Model logits/probabilities	Confidence or reject option	Safer clinical deployment	Not yet standardized	Jung et al. [8], Malinowski et al. [3]

## AI for nuclei detection and segmentation in HER2-SISH

Accurate nuclei detection represents a critical prerequisite for HER2-SISH analysis, as subsequent signal quantification depends on precise identification of nuclear boundaries. Deep learning-based segmentation models have demonstrated significant improvements over traditional image processing methods, particularly in complex tissue environments.

**Fig. 4:**
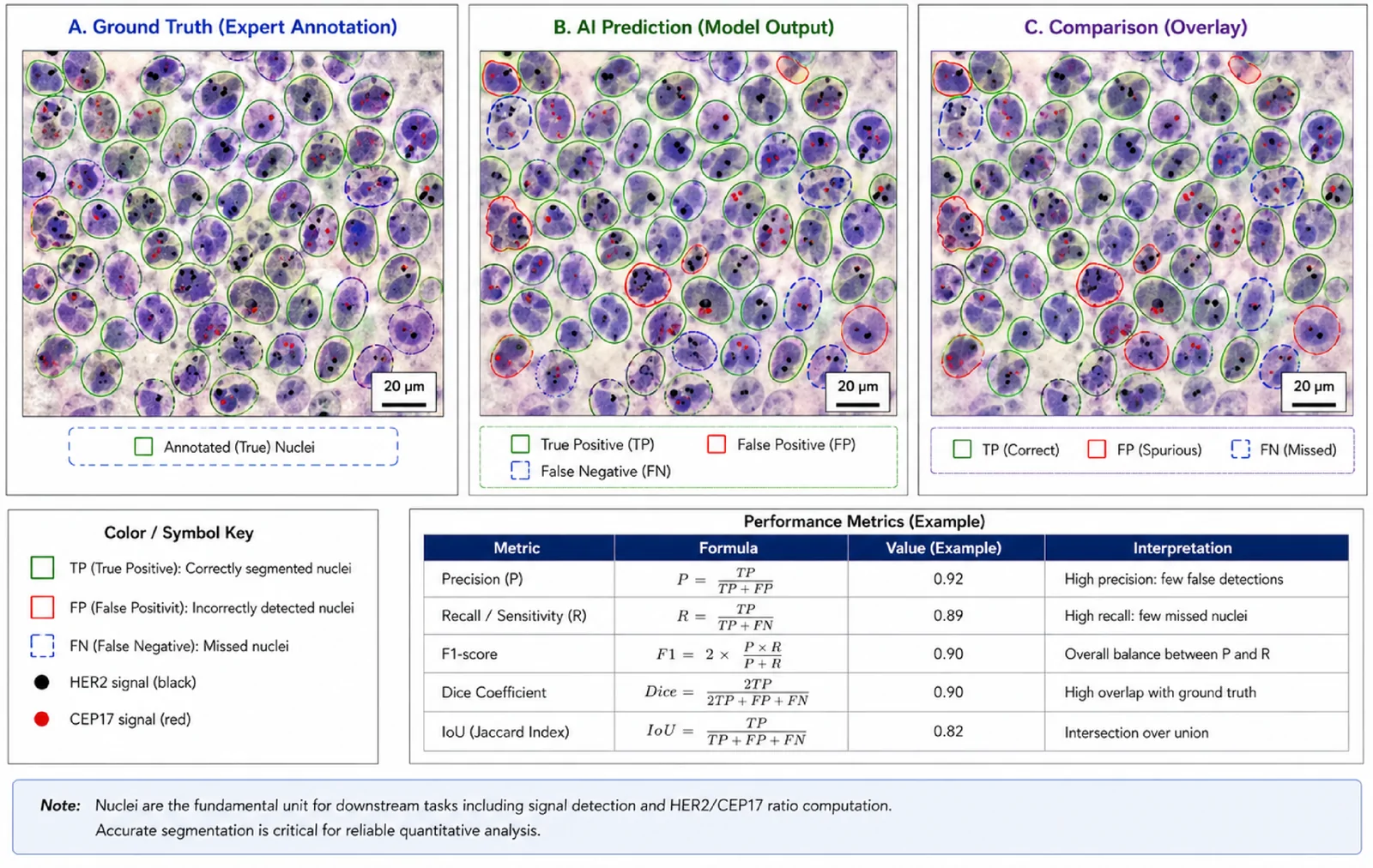
Nuclei segmentation workflow illustrating true positives, false positives, and false negatives

The nuclei segmentation workflow, including classification of true positives, false positives, and false negatives, is illustrated in Fig. 4.

Recent studies have reported high segmentation performance in HER2-SISH images, although these results are influenced by dataset characteristics such as sample size, annotation quality, and scanner variability [14].

Rehman et al. [14] demonstrated that fine-tuned StarDist models trained on expert-annotated regions of interest achieve F1 scores approaching 0.98 in HER2-SISH WSIs. These results are consistent with findings in IHC and H&E domains, suggesting that nuclei segmentation performance is approaching a practical ceiling determined by slide quality rather than algorithmic limitations.

The F1-score, defined as:

$$ F_{1} = \frac{2 \cdot TP}{{2 \cdot TP + FP + FN}} $$

provides a balanced measure of detection accuracy, penalizing both missed nuclei and false detections.

## Automated HER2 and CEP17 signal detection

**Fig. 5:**
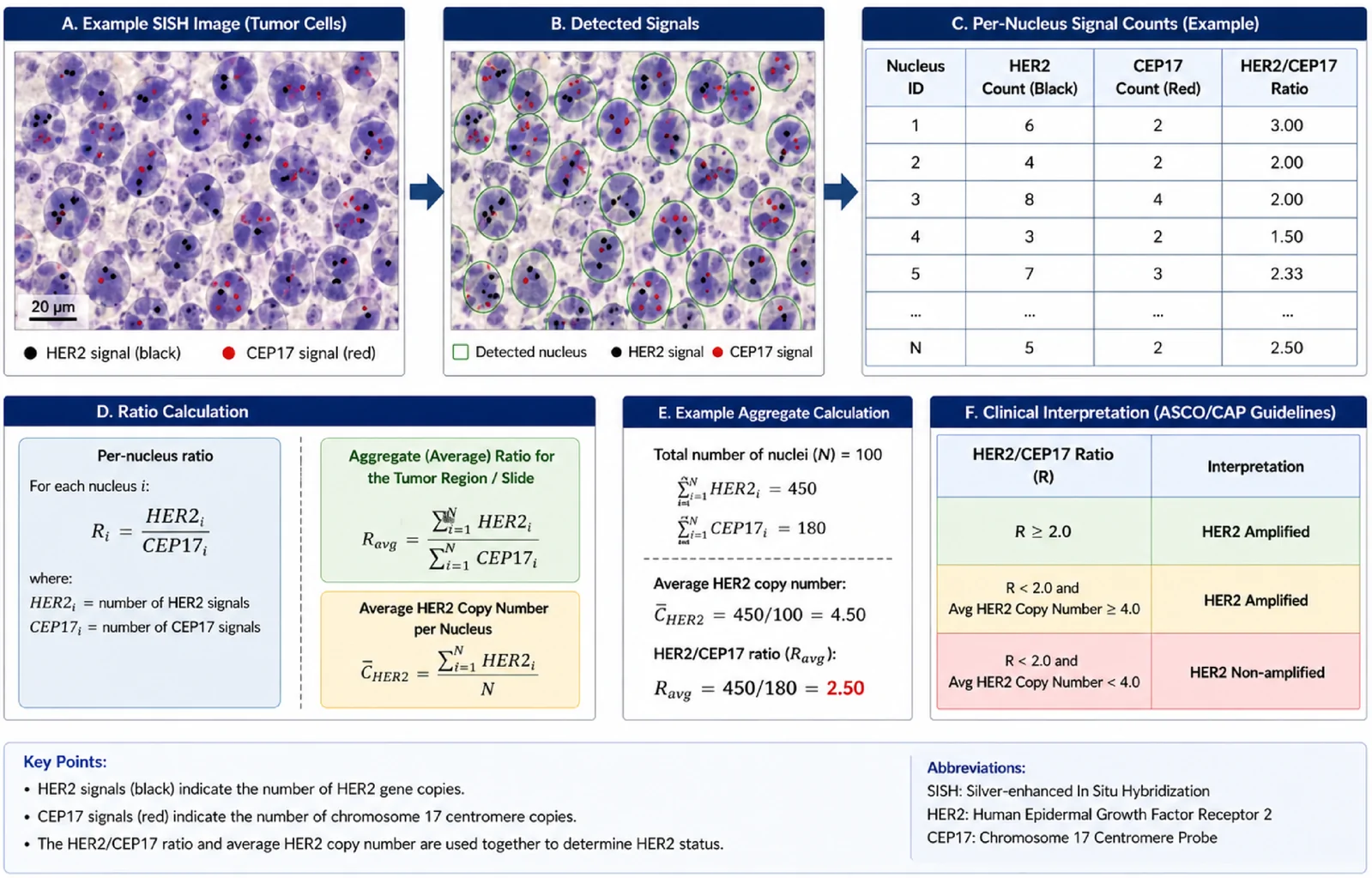
Signal detection and HER2/CEP17 ratio computation workflow

Following nuclei segmentation, artificial intelligence systems must detect HER2 and CEP17 signals and compute clinically relevant metrics. This process is illustrated in Fig. 5.

Signal detection remains challenging due to clustering, overlapping structures, and variability in staining intensity. AI models have demonstrated strong agreement with manual assessment under controlled conditions, although performance may vary in heterogeneous and low-signal cases [13, 15].

Clinical interpretation requires both HER2/CEP17 ratio and average HER2 copy number. Performance benchmarks across tasks and modalities are summarized in Table 4. Table 4 summarizes reported performance benchmarks across AI tasks and HER2 testing modalities. Nuclei segmentation and signal detection tasks demonstrate consistently high technical performance, often exceeding F1-scores of 0.95. Clinically decisive endpoints—such as HER2/CEP17 ratio computation and whole-slide classification—show strong concordance with expert pathologists when algorithms are explicitly aligned with ASCO/CAP guidelines. Performance variability increases in low-signal and heterogeneous cases, underscoring the importance of uncertainty-aware validation strategies.

**Table 4:** Performance benchmarks of artificial intelligence methods for HER2 assessment by task and modality

AI task	Primary modality	Typical performance metrics	Reported performance range	Clinical reference standard	References
Nuclei detection/segmentation	HER2-SISH WSI	Precision, recall, F1-score	F1 ≈ 0.97–0.98	Expert nuclear annotation	Rehman et al. [14], Qaiser et al. [12]
HER2 signal detection	SISH/Dual ISH	Precision, recall	Precision > 0.90	Manual signal counting	Rehman et al. [13], Shi et al. [15]
CEP17 signal detection	SISH/Dual ISH	Precision, recall	Precision > 0.90	Manual signal counting	Shi et al. [15]
HER2/CEP17 ratio computation	Bright-field ISH	Pearson r, concordance (%)	r ≈ 0.90; > 95% concordance	Pathologist ratio (ASCO/CAP)	Rehman et al. [13], Papouchado et al. [10]
Patch-level classification	HER2-SISH	Accuracy, AUC	Acc ≈ 87–99%	Slide-level HER2 status	Tan et al. [17]
Whole-slide classification	HER2-SISH	Accuracy, Cohen’s κ	Acc ≈ 79–98%; κ ≈ 0.70–0.90	Final clinical diagnosis	Tan et al. [17], Qaiser et al. [12]
IHC HER2 scoring (0–3 +)	IHC	Sensitivity, specificity	Sens ≈ 0.95–0.98; Spec ≈ 0.75–0.85	Expert consensus	Albuquerque et al. [1], Wu et al. [19]
HER2-low boundary detection	IHC ± ISH	κ, sensitivity	Improved κ vs manual	Expert consensus	Wu et al. [19], Palm et al. [9]
Multimodal HER2 assessment	IHC + ISH	Accuracy, concordance	Near-expert agreement	Multireader studies	Palm et al. [9]
Uncertainty estimation/QC	SISH/IHC	Calibration error, coverage	Emerging	Human review decision	Jung et al. [8], Malinowski et al. [3]

Performance variability is most evident in borderline and heterogeneous cases, emphasizing the need for uncertainty-aware models and robust validation strategies.

The HER2/CEP17 ratio is computed as:

$$ R = \frac{{\mathop \sum \nolimits_{i = 1}^{N} H_{i} }}{{\mathop \sum \nolimits_{i = 1}^{N} C_{i} }} $$

where $H_{i}$ and $C_{i}$ denote HER2 and CEP17 signals within nucleus *i*, aggregated across *N* tumor nuclei. The full signal aggregation and HER2/CEP17 ratio computation pipeline is visualized in Fig. 5.

## Region-level and whole-slide classification approaches

In addition to signal-based pipelines that explicitly quantify HER2 and CEP17 signals, an alternative methodological paradigm frames HER2 assessment as a classification problem. In this approach, convolutional neural networks or vision transformers are trained to classify image patches or regions as amplified or non-amplified based on learned visual features rather than explicit signal counting. Such approaches have demonstrated high patch-level accuracy in HER2-SISH datasets, as reported by Tan et al. [17], where classification models achieved strong performance under controlled conditions.

However, the clinical relevance of these results depends critically on how patch-level predictions are aggregated to produce slide-level decisions. Whole-slide classification introduces additional complexity due to tumor heterogeneity, uneven signal distribution, and the presence of non-tumor regions. Aggregation strategies such as majority voting, attention-based pooling, and rule-based fusion may produce different outcomes for the same slide, particularly in cases with focal amplification or borderline signal distributions.

**Fig. 6:**
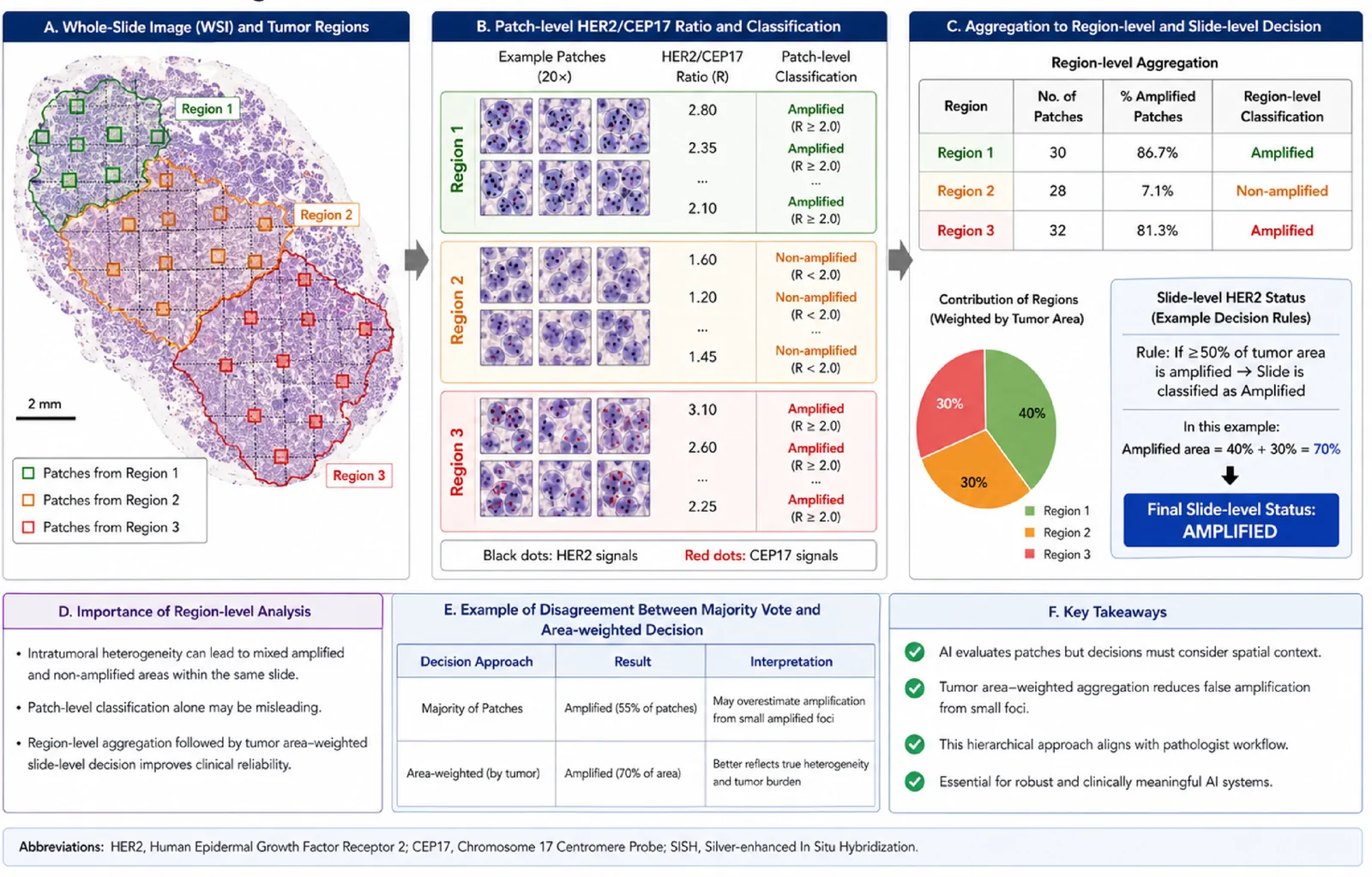
Example segmentation and classification output highlighting patch-level variability and aggregation effects

The variability introduced by aggregation strategies is conceptually illustrated in Fig. 6, which highlights differences between patch-level predictions and whole-slide inference outcomes.

While classification-based approaches offer computational efficiency and reduced reliance on detailed annotations, they raise concerns regarding interpretability and alignment with established diagnostic criteria. Unlike signal-based pipelines, classification models do not inherently provide HER2/CEP17 ratios or copy-number estimates, which are central to clinical HER2 assessment. Consequently, such approaches should be interpreted as complementary tools rather than direct replacements for guideline-aligned workflows.

## Integration with other HER2 modalities

Artificial intelligence applications in HER2 assessment extend beyond SISH to include immunohistochemistry, fluorescence ISH, and dual-color bright-field ISH. These modalities provide important methodological insights that can inform SISH-specific model development, although their results should not be directly extrapolated without careful consideration of modality-specific differences [4, 6].

Studies such as Xue et al. [20] and Zakrzewski et al. [21] have demonstrated reliable HER2 classification in fluorescence ISH images using deep learning, particularly in cases with clear amplification patterns. Similarly, AI-assisted IHC scoring has reached a relatively advanced stage of development, with meta-analyses reporting high sensitivity and moderate specificity in HER2 classification tasks [1]. These approaches have been particularly valuable in reducing interobserver variability in borderline IHC categories, including HER2 0 and 1 + [9, 19].

**Fig. 7:**
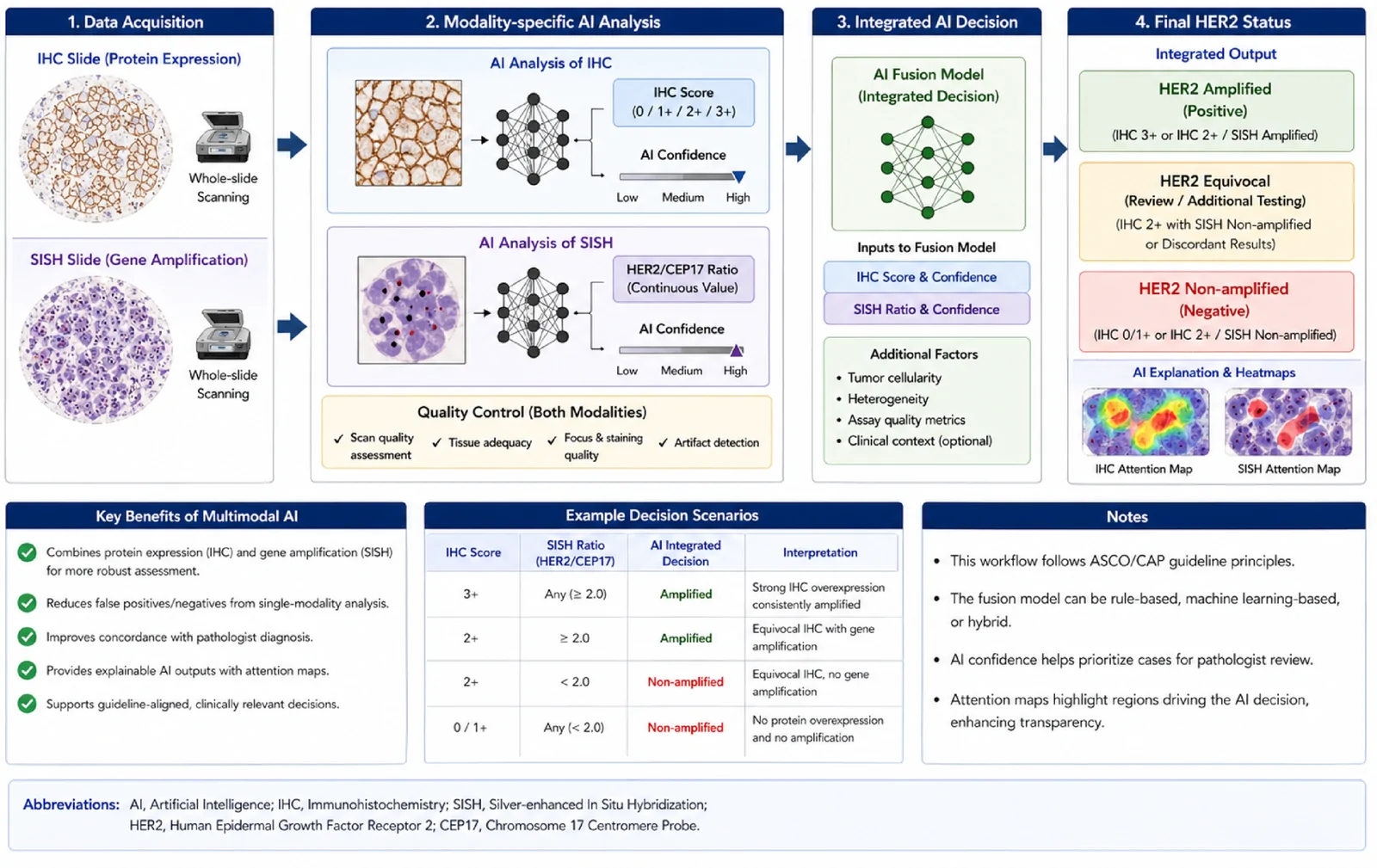
Multimodal integration of AI-assisted IHC and ISH workflows for HER2 assessment

The integration of AI-assisted workflows across IHC and ISH modalities is illustrated in Fig. 7, where multimodal pipelines combine protein expression assessment with gene amplification confirmation.

It is important to emphasize that HER2-low and HER2-ultralow categories remain defined by IHC rather than ISH. Therefore, SISH-based AI systems should not be described as directly determining eligibility for antibody–drug conjugate therapies. Instead, SISH may serve as a complementary modality that provides quantitative gene-level information in selected cases, particularly when integrated with IHC findings.

## Results synthesis: study-by-study mapping (continued and deepened)

A synthesis of the reviewed literature reveals consistent trends across AI-based HER2 assessment studies. Signal-based approaches that explicitly detect HER2 and CEP17 signals and compute ratios tend to demonstrate stronger alignment with clinical reference standards compared with classification-based models. This is primarily because signal-based pipelines replicate the established diagnostic workflow used by pathologists.

Rehman et al. [13] demonstrated that AI-derived HER2/CEP17 ratios showed strong agreement with manual assessment, with no statistically significant differences in final HER2 classification. Similarly, Shi et al. [15] reported that automated analysis reduced variability in ratio estimation, particularly in borderline cases where manual interpretation is most challenging.

In contrast, classification-based approaches, while achieving high patch-level accuracy, often exhibit greater variability at the whole-slide level. This discrepancy highlights the importance of evaluating AI performance in the context of clinically relevant endpoints rather than relying solely on image-level metrics.

**Fig. 8:**
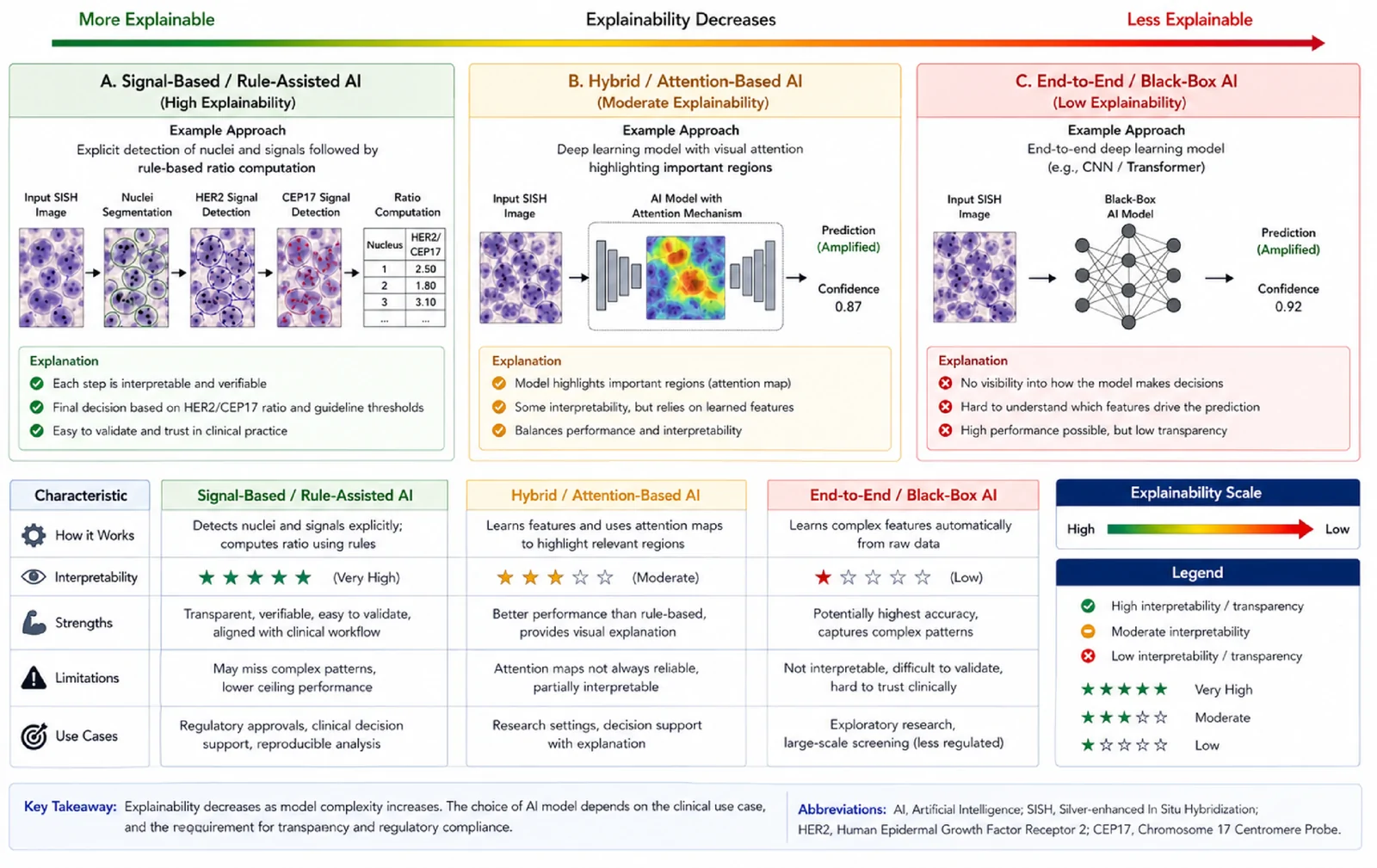
Explainability spectrum for AI approaches in HER2 diagnostics, from signal-based pipelines to end-to-end classification models

The relationship between algorithmic complexity, interpretability, and regulatory readiness is illustrated in Fig. 8.

Signal-based methods occupy a favorable position in this spectrum due to their transparency and direct alignment with clinical guidelines, whereas classification-based methods may achieve high performance but lack interpretability.

The recognition of HER2-low and HER2-ultralow disease as clinically actionable categories has fundamentally altered expectations for HER2 diagnostics. Historically, ISH techniques were optimized for binary amplification detection. However, emerging evidence suggests that sub-amplification patterns and subtle copy-number variations may hold therapeutic relevance, particularly when integrated with IHC findings.

Palm et al. [9] demonstrated that AI-assisted workflows combining IHC and ISH achieve higher agreement in HER2-low categories than either modality alone. While their study primarily focused on IHC, the integration logic is directly applicable to SISH, where quantitative signal distributions can be leveraged to identify borderline cases requiring expert review.

Wu et al. [19] further showed that AI support significantly improves accuracy in distinguishing HER2 0 from 1 + , a distinction that is notoriously difficult even for experienced pathologists. Although this work was conducted in the IHC domain, it highlights a broader principle: AI excels at reducing interobserver variability in low-signal regimes, precisely where human judgment is most challenged.

A critical determinant of translational readiness is performance stability across laboratories, scanners, and staining protocols. While early AI studies were often limited to single-center datasets, more recent investigations have begun to address this limitation [3, 11].

Ring studies and multicenter evaluations, particularly in the IHC domain, consistently demonstrate that AI assistance improves interobserver agreement across institutions [8, 12]. Although fewer multicenter studies exist for SISH specifically, foundational work on SISH reproducibility provides a strong substrate for AI deployment. Papouchado et al. [10] and Dietel et al. [6] showed that SISH itself exhibits low interlaboratory variability, suggesting that AI models trained on diverse SISH datasets may generalize more readily than those trained on fluorescence-based modalities.

Nevertheless, Malinowski et al. [3] emphasized that scanner resolution, focus quality, and illumination uniformity exert a measurable influence on AI performance in bright-field ISH. Their findings indicate that below a minimum quality threshold, algorithmic accuracy deteriorates sharply, irrespective of model sophistication. This observation reinforces the need for standardized pre-analytic quality control in AI-assisted SISH workflows.

## Tumor heterogeneity and focal amplification

Tumor heterogeneity represents one of the most significant challenges in HER2 assessment. Intratumoral variation in gene amplification can result in mixed populations of amplified and non-amplified cells within the same tissue section. This heterogeneity may manifest as focal amplification, where small clusters of tumor cells exhibit high HER2 copy numbers that are not representative of the overall tumor.

Traditional manual assessment accounts for heterogeneity through selective evaluation of representative tumor regions. However, this process is inherently subjective and may overlook small but clinically significant amplified regions. AI systems have the potential to address this limitation by analyzing entire whole-slide images and identifying localized patterns of amplification.

**Fig. 9:**
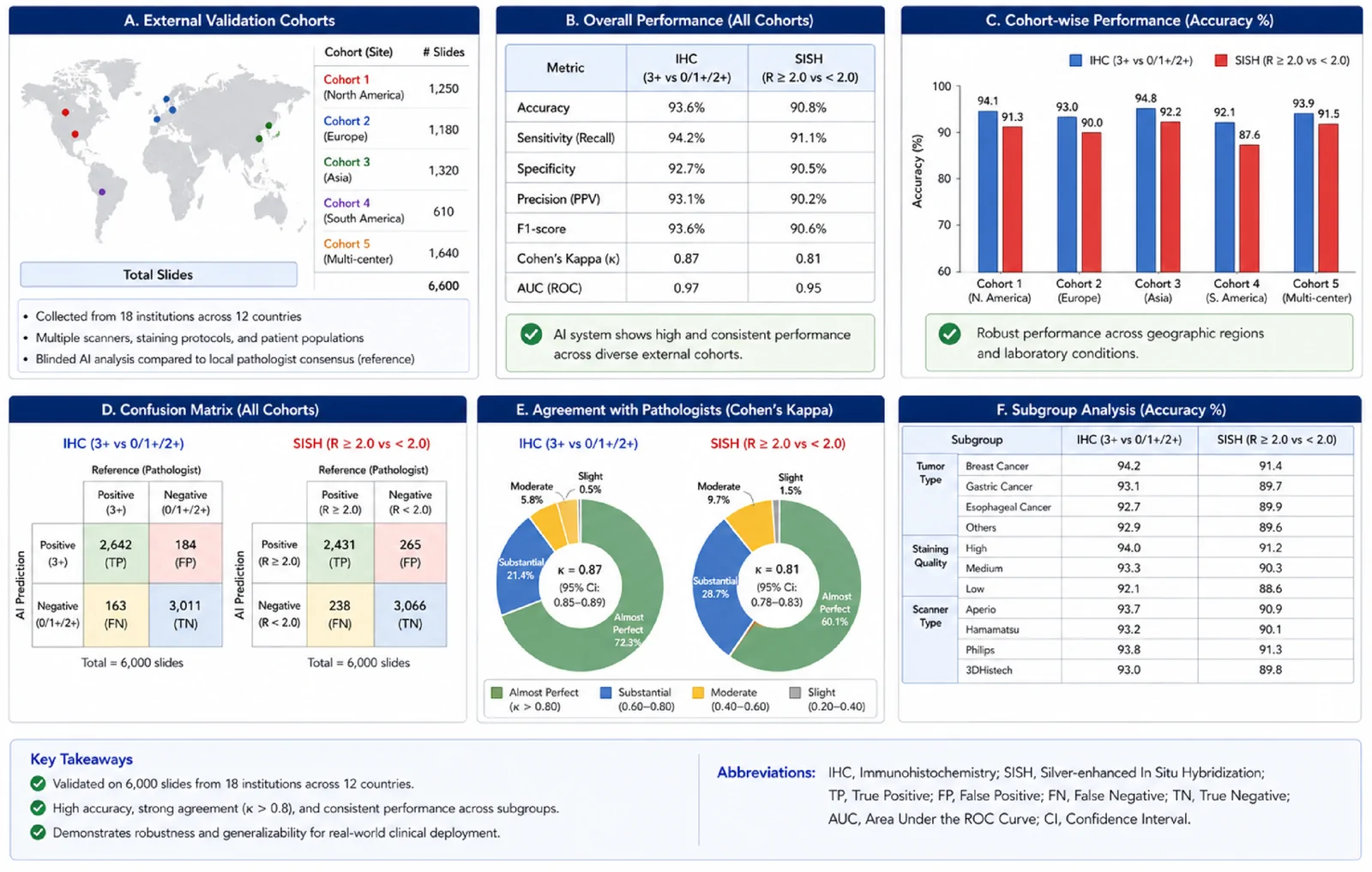
Error sources affecting AI performance in HER2-SISH, including signal clustering, staining variability, and tumor heterogeneity

The impact of heterogeneity on AI performance is illustrated in Fig. 9, which highlights common error sources in HER2-SISH analysis.

However, not all AI approaches are equally suited to handling heterogeneity. Classification-based models that rely on majority voting may effectively average out heterogeneous regions, potentially leading to misclassification of focal amplification. In contrast, signal-based approaches that operate at the nuclei level can capture the distribution of HER2 signals across the tumor, enabling more nuanced analysis.

Recent studies have proposed distribution-aware metrics that report not only mean HER2/CEP17 ratios but also variance, percentiles, and spatial distribution of signals. Such approaches provide a more comprehensive representation of tumor biology and may improve detection of clinically relevant heterogeneity.

## Sources of error and failure modes in HER2-SISH AI systems

AI-based HER2 assessment is subject to multiple sources of error that extend beyond the algorithm itself. Pre-analytical factors such as tissue fixation, section thickness, and staining variability can significantly affect signal appearance. In SISH, these factors may alter the morphology and intensity of silver deposits, leading to misclassification by detection models.

Scanning-related artifacts represent another major source of error. Variations in focus, illumination, and resolution can degrade image quality and reduce the accuracy of AI models. Malinowski et al. [3] demonstrated that suboptimal scanning conditions can significantly impact performance in bright-field ISH analysis.

**Fig. 10:**
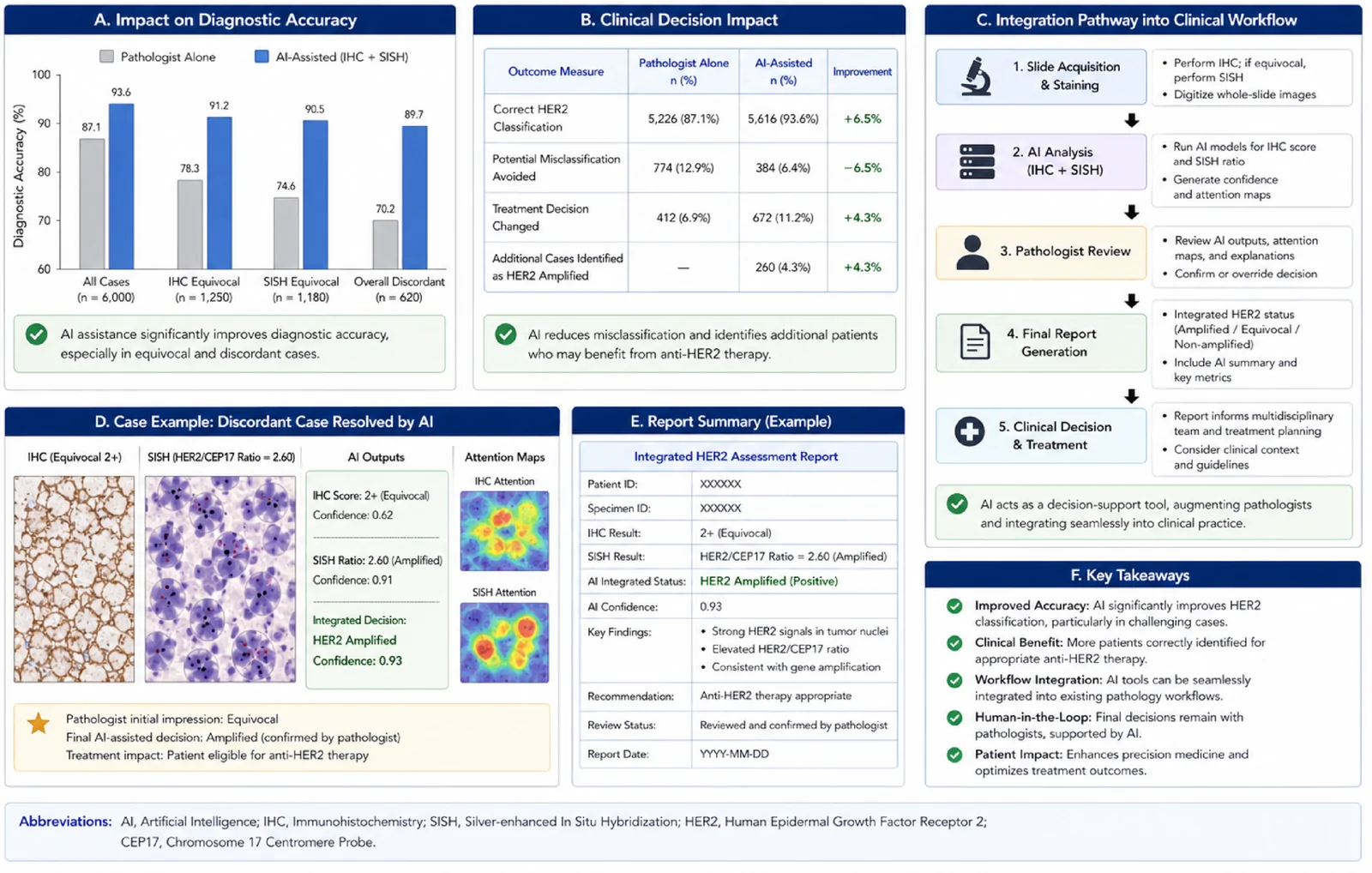
Clinical impact and integration pathway of AI-assisted HER2 assessment

The relationship between scanning quality and AI performance is illustrated in Fig. 10.

Annotation bias also plays a critical role in model performance. Training datasets often rely on expert-selected regions of interest, which may preferentially include areas with clear amplification while underrepresenting subtle or borderline cases. This can result in models that perform well on curated datasets but fail to generalize to real-world conditions.

Additionally, domain shift across laboratories, including differences in staining protocols, scanner hardware, and patient populations, may further limit generalizability. These factors underscore the importance of external validation and multicenter studies in evaluating AI systems.

## Critical appraisal of current evidence

Despite encouraging results, the current evidence base for AI-assisted HER2-SISH assessment remains limited in several important respects. Many studies are based on relatively small datasets, often consisting of fewer than a few hundred whole-slide images. Such sample sizes may be insufficient to capture the full range of biological and technical variability encountered in clinical practice.

Furthermore, a significant proportion of studies rely on retrospective data collected under controlled conditions. While these studies provide valuable insights into algorithm performance, they do not fully reflect the complexity of real-world clinical environments. External validation across multiple institutions remains limited, particularly for SISH-specific datasets.

Ground truth definitions also vary across studies. Some investigations use expert annotations at the nuclei or signal level, while others rely on slide-level HER2 status derived from clinical reports. These differences complicate direct comparison of results and highlight the need for standardized evaluation protocols.

Another limitation is the tendency to report performance using generalized metrics such as accuracy or concordance without sufficient contextual information. For example, high accuracy in patch-level classification does not necessarily translate into reliable slide-level HER2 classification, particularly in heterogeneous tumors.

The limitations of current datasets and validation strategies are summarized in Table 5. Table 5 shows Most AI studies in HER2-SISH remain single-center, with controlled scanning conditions. However, the biological robustness of SISH itself, demonstrated in earlier multicenter validation studies, provides a strong foundation for future multi-institutional AI deployment.

**Table 5:** Dataset and validation characteristics of AI studies for HER2 assessment using SISH and related modalities

References	Year	Modality	Dataset size (Slides/Cases)	Cancer type	Single vs. multicenter	Scanner/Stain diversity	Validation strategy
Dietel et al. [6]	2007	SISH vs FISH	245 slides	Breast	Multicenter	Multiple labs, SISH/FISH	Inter-lab concordance
Papouchado et al. [10]	2010	SISH	200 + cases	Breast	Multicenter	Multiple scanners	Interobserver reproducibility
Sung et al. [16]	2010	SISH/IHC	110 cases	Breast	Single center	Limited scanner diversity	Method comparison
Rehman et al. [13]	2024	HER2-SISH WSI	~ 120 WSIs	Breast	Single center	Single scanner, controlled stain	Train/val/test split
Rehman et al. [14]	2025	HER2-SISH WSI	~ 150 WSIs (ROI-based)	Breast	Single center	Bright-field SISH	Cross-ROI validation
Shi et al. [15]	2024	Dual bright-field ISH	180 cases	Breast	Multicenter	Multiple scanners	External clinical validation
Tan et al. [17]	2023	HER2-SISH	~ 90 WSIs	Breast	Single center	Limited diversity	Patch-level + WSI aggregation
Palm et al. [9]	2023	IHC + ISH	300 + cases	Breast (primary & metastatic)	Multicenter	Multiple clones & scanners	Reader study + AI comparison
Wu et al. [19]	2023	IHC	400 + slides	Breast	Multicenter	High stain variability	External test cohort
Xue et al. [20]	2023	FISH	250 + images	Breast	Single center	Fluorescence only	Cross-validation

These observations underscore the need for more rigorous study design, including larger datasets, multicenter validation, standardized reporting, and prospective clinical evaluation.

## Reproducibility, data availability, and benchmarking

Reproducibility represents a critical challenge in AI-based HER2 assessment. The scarcity of publicly available SISH datasets limits the ability of researchers to validate and compare models across studies. Unlike IHC and general histopathology datasets, which have benefited from open benchmarks and competitions, SISH datasets remain largely proprietary.

The lack of standardized datasets also complicates regulatory evaluation, as performance claims cannot be independently verified. Initiatives such as the HER2 Challenge Contest [12] have demonstrated the value of shared benchmarks in advancing the field, but similar efforts are needed for SISH-specific applications.

The relationship between dataset characteristics, validation strategies, and clinical alignment is summarized in Table 6. Table 6 shows Validation strategies that emphasize clinical concordance and guideline-aligned outputs (e.g., HER2/CEP17 ratio) demonstrate stronger translational relevance than purely image-centric metrics.

**Table 6:** Validation endpoints, metrics, and clinical alignment in AI-based HER2 assessment

AI task	Primary metric(s)	Clinical comparator	Clinical alignment	Typical pitfalls
Nuclei detection/segmentation	Precision, Recall, F1-score	Expert nuclear annotation	Indirect (enables downstream tasks)	Over-segmentation in dense regions
HER2 signal detection	Precision, Recall	Manual dot counting	Direct	Signal clustering, noise
CEP17 signal detection	Precision, Recall	Manual dot counting	Direct	Misclassification of background
HER2/CEP17 ratio computation	Pearson r, Concordance (%)	Pathologist ratio	Very high (ASCO/CAP compliant)	Ratio instability in low counts
Patch-level classification	Accuracy, AUC	Slide-level HER2 status	Moderate	Loss of interpretability
WSI-level aggregation	Accuracy, Kappa	Final clinical diagnosis	High (if rule-based)	Sensitivity to heterogeneity
HER2-low boundary detection	Kappa, Sensitivity	Expert consensus	Increasing importance	Subjective ground truth
Uncertainty estimation	Coverage, Calibration error	Human review decision	Emerging	Poorly standardized

Future research should prioritize the development of publicly available datasets, standardized evaluation protocols, and collaborative benchmarking initiatives to improve reproducibility and accelerate clinical translation.

## Regulatory landscape and translational readiness

The translation of artificial intelligence systems for HER2 assessment from research settings into clinical practice requires compliance with regulatory frameworks governing software as a medical device. Regulatory agencies such as the United States Food and Drug Administration and European regulatory bodies require evidence of analytical validity, clinical validity, and clinical utility before approval of AI-based diagnostic systems.

In the context of HER2 assessment, signal-based AI systems developed for SISH analysis have a relative advantage compared with purely classification-based approaches. This advantage arises from the fact that signal-based systems produce outputs that are directly aligned with established clinical workflows, including HER2 signal counts, CEP17 signal counts, HER2/CEP17 ratios, and average HER2 copy number. These outputs correspond to the parameters already used by pathologists in routine practice, thereby facilitating interpretability and regulatory acceptance.

**Fig. 11:**
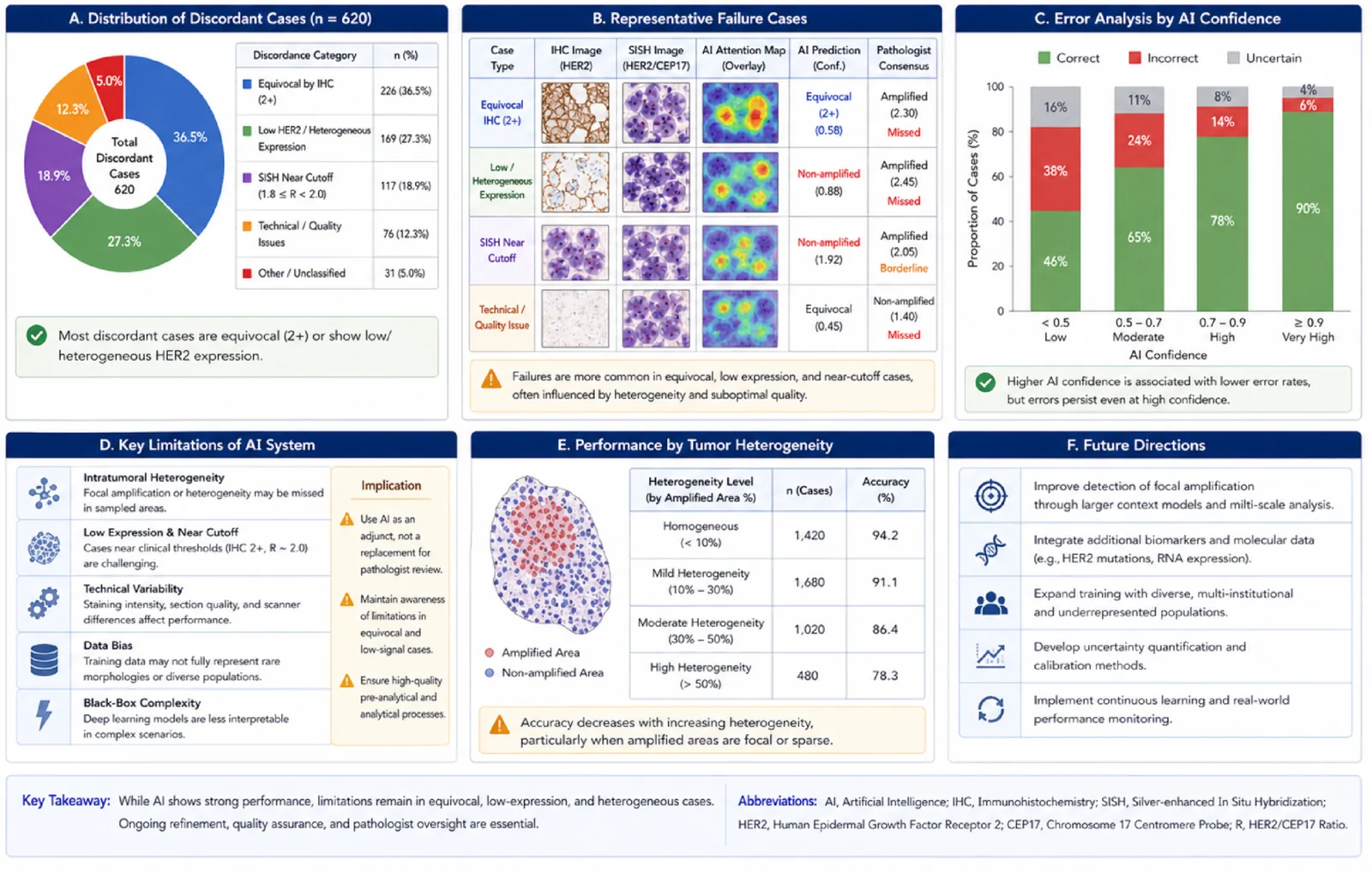
Failure analysis and limitations of AI-assisted HER2 assessment

**Fig. 12:**
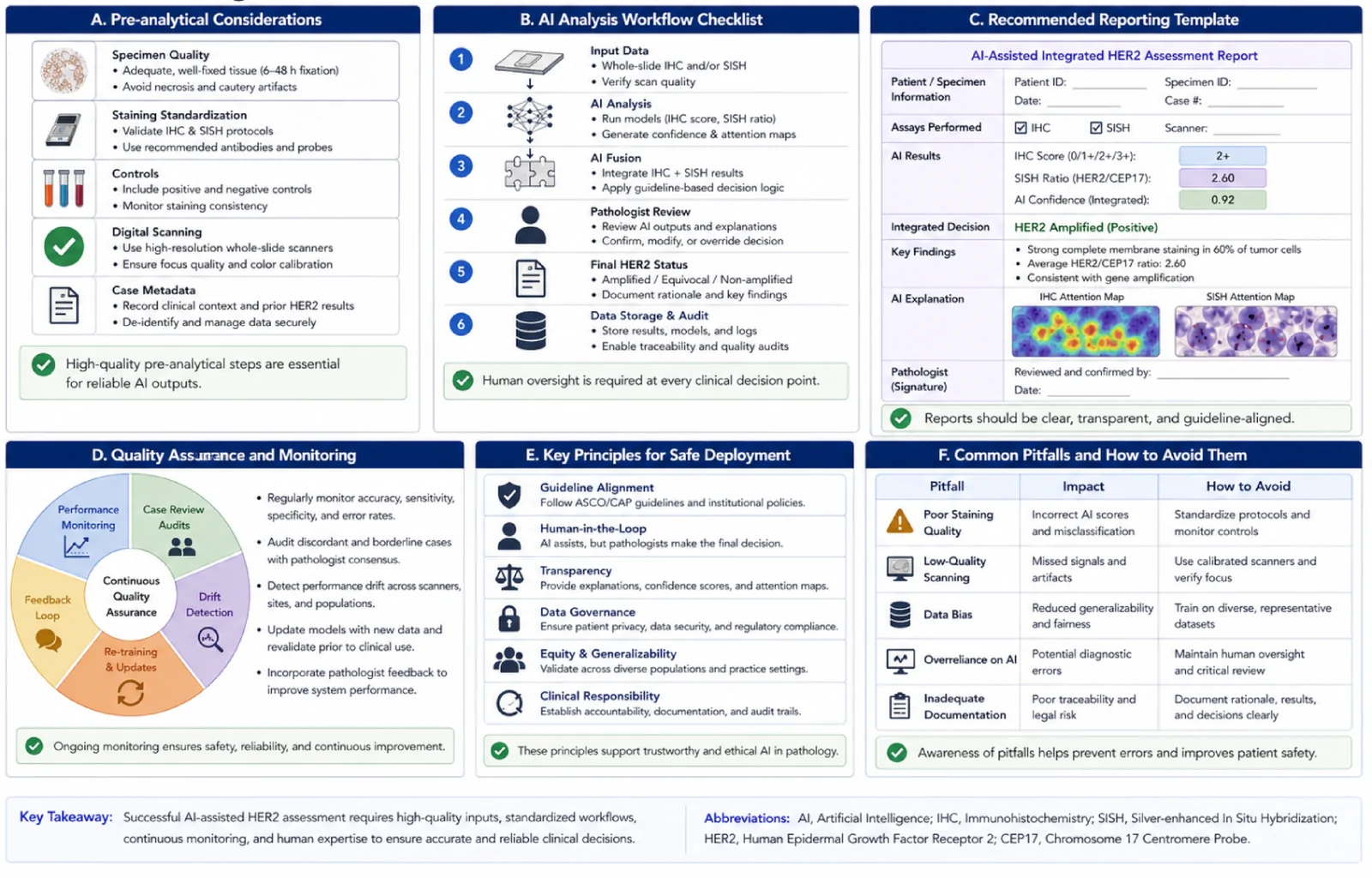
Guidelines and best practices for AI-assisted HER2 assessment

**Fig. 13:**
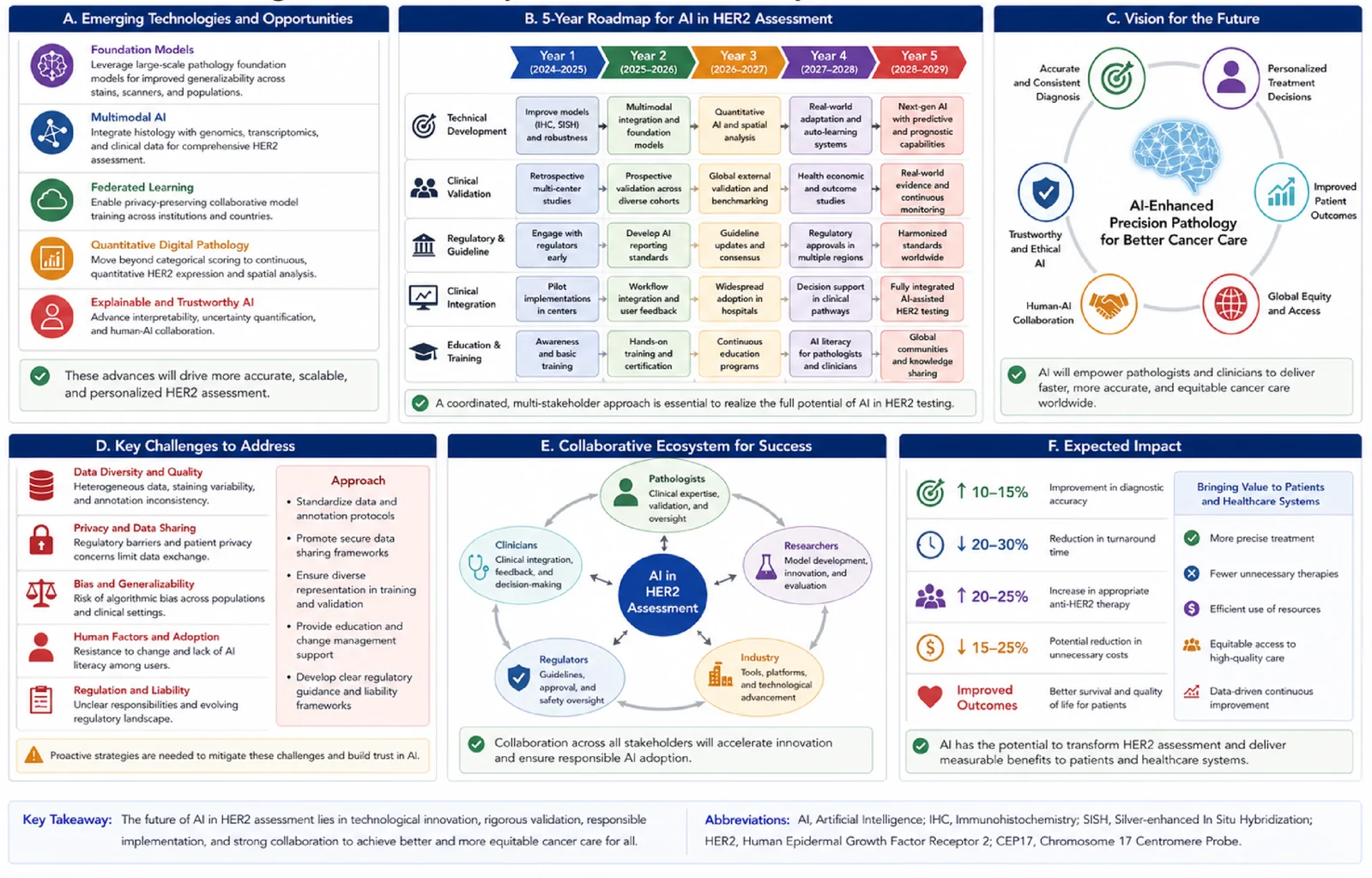
Future perspectives and roadmap for AI in HER2 assessment

Representative failure modes and limitations of AI-assisted HER2 assessment are summarized in Fig. 11. Recommended guidelines and best practices for AI-assisted HER2 assessment are illustrated in Fig. 12. Emerging trends, future directions, and translational roadmaps for AI-assisted HER2 assessment are summarized in Fig. 13.

In contrast, end-to-end classification models must demonstrate not only predictive accuracy but also explainability and reproducibility across diverse clinical environments. The lack of explicit intermediate outputs in such models presents challenges for regulatory evaluation, particularly in cases where decision logic cannot be easily interpreted.

An additional consideration in regulatory approval is the requirement for real-world performance monitoring. AI systems deployed in clinical settings must be continuously evaluated to detect performance drift resulting from changes in staining protocols, scanner hardware, or patient populations. This requirement highlights the importance of robust validation strategies and post-deployment monitoring frameworks.

**Fig. 14:**
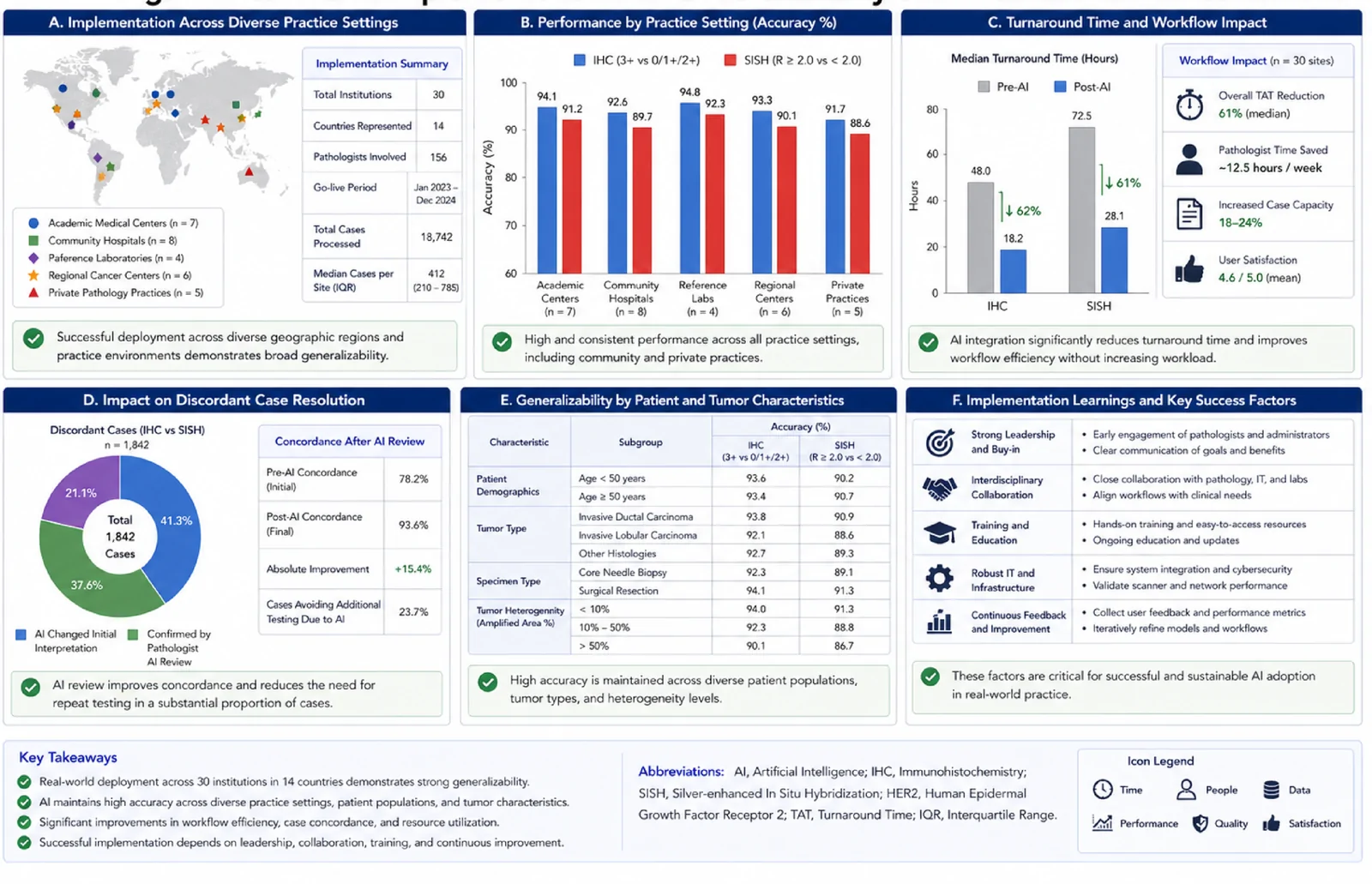
Real-world implementation and generalizability of AI-assisted HER2 assessment

Uncertainty-aware AI models offer a promising approach to improving clinical safety by identifying cases that require human review. The real-world implementation and generalizability of AI-assisted HER2 assessment across diverse practice settings are illustrated in Fig. 14.

Such models categorize outputs into high-confidence predictions, low-confidence cases requiring pathologist review, and cases that should be rejected due to insufficient image quality or ambiguous signals. This approach aligns with regulatory expectations for safe and interpretable AI deployment.

## Ethical considerations and explainability

The integration of artificial intelligence into HER2 diagnostics raises important ethical considerations, particularly regarding transparency, accountability, and the risk of automation bias. Automation bias refers to the tendency of users to over-rely on algorithmic outputs, potentially leading to errors if incorrect predictions are accepted without critical evaluation.

In HER2 assessment, where diagnostic decisions directly influence treatment selection, the consequences of such errors can be significant. Therefore, AI systems must be designed as decision-support tools that augment, rather than replace, expert judgment. This principle is consistently emphasized across the literature and is supported by studies demonstrating improved interobserver agreement when AI is used as an assistive tool [8].

Explainability plays a central role in mitigating these risks. Signal-based SISH AI systems offer inherent explainability by providing intermediate outputs such as detected nuclei, signal counts, and ratio calculations. These outputs allow pathologists to verify the algorithm’s reasoning and maintain control over the diagnostic process.

While classification-based models may achieve high predictive performance, their reliance on abstract feature representations can limit interpretability. Visualization techniques such as attention maps provide partial insight but do not always correspond to biologically meaningful features. As a result, regulatory and clinical adoption is likely to favor models that provide transparent and interpretable outputs.

## Clinical implications and workflow integration

From a clinical perspective, AI-assisted HER2-SISH analysis offers several potential benefits, including reduction of interobserver variability, improved reproducibility, and increased efficiency in high-volume pathology laboratories. These advantages are particularly relevant in settings with limited access to specialized expertise.

The clinical benefits and limitations of AI-assisted HER2 assessment are summarized in Table 7. Table 7 shows AI-assisted SISH analysis offers clear operational and diagnostic advantages, but its safe clinical deployment requires robust quality control, uncertainty awareness, and continued human oversight.

**Table 7:** Clinical benefits and limitations of AI-assisted HER2 assessment using SISH

Clinical benefits	Limitations and challenges
Reduced interobserver variability	Limited multicenter training data
Objective HER2/CEP17 ratio computation	Sensitivity to scanning quality
High reproducibility across nuclei counts	Performance drop in rare ISH groups
Ability to analyze thousands of nuclei	Dependence on annotation quality
Improved consistency in borderline cases	Domain shift across laboratories
Support for HER2-low / ultralow assessment	Lack of standardized QC thresholds
Time and workload reduction for pathologists	Regulatory approval complexity
Transparent, explainable outputs (signal-based)	Risk of automation bias
Scalable deployment in high-volume labs	Integration with LIS/WMS systems

AI systems can assist pathologists by pre-screening slides, highlighting regions of interest, and providing quantitative measurements. However, successful integration into clinical workflows requires careful consideration of usability, interoperability with laboratory information systems, and training of personnel.

Importantly, AI outputs should be presented in a manner that supports decision-making without overwhelming the user. Interfaces that display intermediate results, such as detected nuclei and signal distributions, can enhance interpretability and facilitate adoption.

## Limitations of the current evidence base

Despite the promising potential of AI-assisted HER2-SISH analysis, several limitations must be acknowledged. The majority of studies are based on retrospective datasets collected under controlled conditions, which may not fully represent the variability encountered in clinical practice. Dataset sizes are often limited, and external validation across multiple institutions remains relatively rare.

Another limitation is the lack of standardized datasets and benchmarking protocols. The scarcity of publicly available SISH datasets restricts reproducibility and hinders comparison between different AI approaches. This challenge is particularly significant in contrast to other domains of computational pathology, where open datasets have accelerated methodological development.

Performance reporting also varies across studies, with some investigations emphasizing technical metrics such as accuracy or F1-score without sufficient consideration of clinical endpoints. As discussed earlier, clinically meaningful evaluation requires assessment of HER2 status concordance, particularly in borderline and heterogeneous cases.

These limitations highlight the need for more rigorous study design, including multicenter validation, standardized reporting, and prospective clinical trials.

## Future directions and research priorities

Future research in AI-assisted HER2 assessment is likely to focus on improving robustness, generalizability, and clinical integration. One promising direction is the development of distribution-aware models that capture not only mean HER2 values but also spatial and statistical characteristics of signal distributions within tumors.

The integration of multimodal data, including IHC, SISH, H&E morphology, and genomic information, represents another important avenue for research. Such approaches may enable more comprehensive characterization of tumor biology and support personalized treatment strategies.

The establishment of standardized datasets and benchmarking initiatives will be critical for advancing the field. Collaborative efforts similar to the HER2 Challenge Contest [12] could facilitate comparison of methods and promote reproducibility.

Finally, prospective clinical studies are needed to evaluate the real-world impact of AI-assisted HER2 assessment on diagnostic accuracy, workflow efficiency, and patient outcomes.

## Conclusion

Artificial intelligence has reached a level of maturity that enables meaningful support for HER2 assessment using silver-enhanced in situ hybridization and related bright-field ISH modalities. By automating tasks such as nuclei detection, signal quantification, and HER2/CEP17 ratio computation, AI systems have the potential to reduce variability and improve efficiency in diagnostic workflows.

However, the current evidence base remains limited by methodological constraints, including small datasets, retrospective designs, and limited external validation. Performance claims must therefore be interpreted with caution, particularly in heterogeneous and borderline cases where diagnostic accuracy is most critical.

When implemented as transparent, uncertainty-aware decision-support tools, AI systems can enhance rather than replace the role of the pathologist. Future progress will depend on rigorous validation, standardized datasets, and close collaboration between clinicians, pathologists, and AI researchers.

The convergence of digital pathology, artificial intelligence, and clinical oncology suggests that AI-assisted HER2 assessment is well positioned to transition from experimental research to routine clinical practice, provided that its development is guided by principles of transparency, reliability, and clinical relevance.

## Data Availability

No new data were generated or analyzed. The evidence considered is contained in the cited publications.
